# Riboflavin Arrests Cisplatin-Induced Neurotoxicity by Ameliorating Cellular Damage in Dorsal Root Ganglion Cells

**DOI:** 10.1155/2015/603543

**Published:** 2015-12-02

**Authors:** Maria Salman, Imrana Naseem, Iftekhar Hassan, Aijaz A. Khan, Ibrahim M. Alhazza

**Affiliations:** ^1^Department of Biochemistry, Faculty of Life Sciences, Aligarh Muslim University, Aligarh 202002, India; ^2^Department of Zoology, College of Sciences, King Saud University, Riyadh 11451, Saudi Arabia; ^3^Department of Anatomy, JNMCH, Aligarh Muslim University, Aligarh 202002, India

## Abstract

Cis-Diamminedichloroplatinum II- (CP-) induced neurotoxicity is one of the least explored aspects of this drug. Dorsal root ganglia (DRG) cells are considered as the primary target, and their damage plays a vital role in pathogenesis and etiology of CP-induced neurotoxicity. The present study is aimed at confirming if riboflavin (RF) has any protective role in shielding the DRG from CP-induced toxicity. After conducting the established treatment strategy on mice under photoillumination, it was observed that, despite the fact that RF alone is partially toxic, its combination with CP significantly ameliorated the drug-induced damage in DRG cells as evidenced by histological analysis. In addition, it was interesting to observe that the combination group (RF + CP) was able to induce apoptosis in the target cells up to a significant extent which is considered as the most preferred way of countering cancer cells. Therefore, RF can act as an effective adjuvant compound in CP-based chemoradiotherapy to improve clinical outcomes in the contemporary anticancer treatment regimes.

## 1. Introduction

Cis-Diamminedichloroplatinum II (CP) is one of the most prominent antineoplastic agents for the treatment of various forms of cancers and solid tumors. Despite being effective, it exerts serious side effects that limit its optimized clinical application. Neurotoxicity is one of the least studied toxic aspects of this drug that makes the patients undergoing chemotherapy suffer from clinical as well as social issues [1, 2]. The neurotoxicity primarily involves peripheral neuropathy evidenced by neuropathic pain, sensory impairment, sensory ataxia, demyelination, and axonal degeneration of the neurons. Its severity is directly proportional to the cumulative dose of the drug and duration of the chemotherapy [3].

A large number of the reports show that DNA-CP complex products are intensely located in the target nerve cells like in sensory cells and dorsal root ganglion (DRG) cells leading to various pathological conditions related to nervous system after CP-based chemotherapy [4]. Besides, CP also generates reactive oxygen species (ROS) and reactive nitrogen species (RNS) upon accumulating in the target cells that can alter normal physiological as well as redox status of the cells resulting in many deleterious effects including the drug-induced neurotoxicity in neuronal and allied cells [5, 6].

Riboflavin (RF) or vitamin B_2_ participates in various enzyme-catalyzed redox metabolic reactions in the form of flavin adenine dinucleotide (FAD) and flavin mononucleotide (FMN). Furthermore, an efficient photosensitizer generates free radicals like riboflavin radicals, superoxide anions, and hydroxyl radicals [7, 8]. This property of RF has been utilized for ribophototherapy and photodynamic therapy effectively both in intact manner and in its photoactivated form [9, 10]. Its deficiency has been manifested to increase the susceptibility of animals and humans to various diseases including cancer; however, its effect on the efficacy of anticancer drugs like methotrexate* in vivo* has been debatable [11]. Earlier, our lab has shown how RF can blunt the toxic insults of CP in mice keratinocytes under photoillumination [12]. Later, we also observed its alleviative effect on the CP-induced neurotoxicity equally effective* in vivo* [13]. As most of the investigators believe that DRG is the main target and their damage triggers CP-induced neurotoxicity, the current study is aimed at confirming the ameliorative efficacy of RF in DRG region of the male mice evidenced by their histopathological investigation.

## 2. Materials and Methods

### 2.1. Chemicals

Riboflavin (CAS number 83-88-5) and cisplatin (CAS number 15663-27-1) were purchased from Sigma-Aldrich Chemical Company, St. Louis, USA. Hematoxylin and Eosin staining was bought from Qualigens Fine Chemicals, Mumbai, India. The rest of all other chemicals used in this work were of analytical grade.

### 2.2. Animal Treatment and Sample Preparation

Twenty-four adult Swiss albino healthy male mice aged 6 months and weighing 35–40 g were used in the present study approved by the Departmental Ethical Committee as in the earlier studies. The mice were acclimatized for a week before starting the treatment. They were sheltered in sufficiently large cages with well-maintained room temperature and 12-hour day-night cycle. They were fed standard pellet mice diet and clean drinking water* ad libitum*.

All the animals were randomly divided into four groups (6 mice/group). They were designated as group I (control), group II (riboflavin (RF, 2 mg/kg body weight) treated), group III (cisplatin (CP, 2 mg/kg body weight) treated), and group IV which was given the combination of cisplatin and riboflavin (2 mg/kg body weight of CP with 2 mg/kg body weight of RF), respectively. All the doses of the test chemicals were administered to the animals intraperitoneally with a 1 mL syringe as per our standardized dose and treatment regime. All the chemicals were dissolved in saline. RF was administered 30 minutes prior to CP treatment in combination treated group IV. All the groups were mildly shaved on their dorsal surface and were kept under full body irradiation by Philips fluorescent light (fluence rate = 38.6 w/m^2^) at 10 cm distance during the daytime with the beginning of the treatment. The mice were given a daily injection for three days, followed by its repetition in every other week and finally three more daily injections to mimic the current treatment strategy implemented for the cancer patients. After completion of the treatment, all the animals were sacrificed on the same day.

### 2.3. Isolation of DRG Cells from the Treated Mice

After an overdose of chloroform anesthesia, the animals were fixed by intracardiac perfusion method with Karnovsky's fixative. The cervical part of the spinal cord and associated root ganglia were dissected from dorsal aspect by laminectomy. The rootlets of spinal nerves were identified and with the help of fine forceps the DRG were pulled out gently from the intervertebral foramina, collected, and processed for paraffin embedding [14, 15].

### 2.4. Processing of DRG Cells for Histopathological Study

From immersion fixed tissue in Karnovsky's fixative, they were trimmed to small blocks containing the ganglia and processed for paraffin embedding. 7 *µ*m thick sections were cut by rotatory microtome followed by their staining with Hematoxylin and Eosin. The prepared sections were visualized under the light microscope (Olympus, BX40, Japan) and interesting representative photomicrographs were taken with the high power integrated camera (X40 objective).

### 2.5. Manual Counting of Cells under the Microscope

Five histological slides were randomly selected from each group of ganglia, which were subjected to counting of cells under light microscopy manually blinded to the treatment. The cells that were showing normal shape and size with a clearly well-defined nucleus and intact cytoplasm were considered as normal cells. The cells exhibiting nuclear shrinkage or its partial/complete disappearance were considered as apoptotic cells whereas cells showing fuzzy nucleus and scattered cytoplasm were assumed as the cells undergoing necrosis [16].

## 3. Results

It is documented that CP causes peripheral neuropathy targeting principally in the DRG cells, so the present study was focused on the histopathological examination of DRG cells only. Histopathology is one of the most reliable technologies to demonstrate the exact effect of any treatment on the target tissue(s). In the present work, the CP treated animals demonstrated typical observational symptoms of peripheral neuropathy that were obvious from their slowing down, losing body weight, imbalance while moving in the cage, and late response in food and maze experiments.

**Figure 1:**
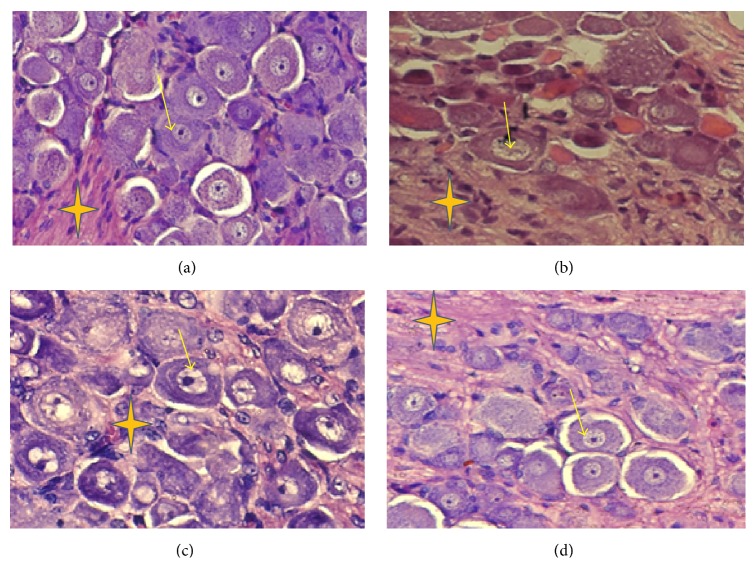
Sample photomicrographs from the thoracic dorsal root ganglia of mice. Control group (a) shows pseudounipolar neuron with centrally placed nucleus and prominent nucleolus (↑) while cisplatin treated group (b) shows relatively small shrunken neuron, smaller nucleolus, and rarified bundle of nerve fibers (N) and prominent neuroglial cells. Riboflavin treated (c) and combination treated (d) groups show neuronal sizes and nucleolar features comparable to control group. All tissue sections were prepared with H&E stain followed by snapping at magnification of 400x. Arrows indicate neurons while stars indicate the nerve fibres.

The control, group I (Figure 1(a)), showed the features of normal cellular structure of DRG like pseudounipolar neuron with a centrally placed nucleus, prominent nucleolus, and soma surrounded by satellite cells (↑) and nerve fascicle (N). Similar histopathological details were observed in RF treated group II slide (Figure 1(c)) showing marginal differences from the control. However, CP treated, group III (Figure 1(b)) exhibited relatively small shrunken neurons with smaller nucleolus, a rarefied bundle of nerve fibers (N), and prominent neuroglial cells. These are the hallmark features of extensive cellular damage, and many of the cells showed necrotic characteristics like swollen cells with scattered cytoplasm. Interestingly, the combination of CP with RF, group IV showed normal neuronal sizes and nucleolar features that are indicative of the restoration of histopathological details of DRG cells; otherwise the cell debris demonstrating apoptotic features was also observed in the same group (Figure 1(d)).

## 4. Discussion

**Figure 2:**
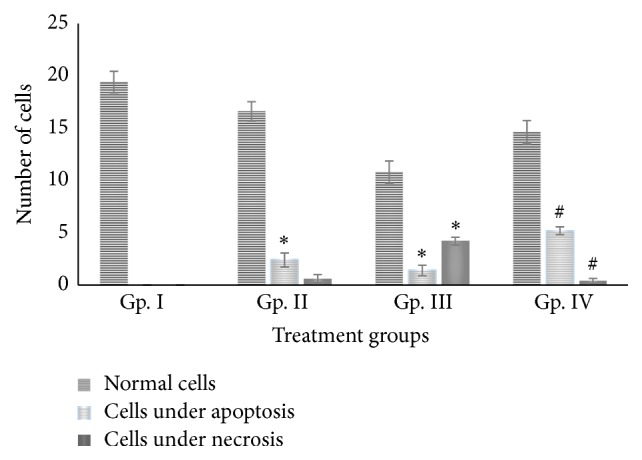
Showing bar graph of cells undergoing necrosis and apoptosis after the treatment with riboflavin, cisplatin, and their combination in dorsal root ganglia of mice. **∗** indicates significant difference from group I (control) at *p* ≤ 0.05.** # **indicates significant difference from group III at *p* ≤ 0.05.

It is well established that CP elicits its neurotoxicity by elevating oxidative and nitrosative stress that disturb the structural and functional homeostasis in the neuronal cells [13, 17, 18]. This is quite evident from Figure 1(b), where the cellular structure of DRG cells in CP treated group showed classic features of necrosis in most of the cells besides apoptosis in few of the other cells. Manual counting under the microscope further showed that CP caused necrosis in 25.6% of the total cells while only 8.5% of the cells exhibited apoptotic features (Figure 2). Similarly, RF treatment led to apoptosis in 12.2% and necrosis in 3% of the total cells. However, the combination of CP with RF showed a significant increase in apoptosis by 27% while necrosis was observed in merely 2% of the total cell count (Figure 2).

It is documented that the extent of oxidative stress and level of energy in any cell decide the fate of that cell if it will progress to successive cell division or will die by undergoing apoptosis or necrosis. If the stress level inside the cell is beyond a certain threshold level with the high-energy deficit, the cell undergoes necrosis but if the stress is moderate with moderate level of energy, the cell follows cell death by apoptosis [6, 16, 19]. Earlier, it has been reported that CP binds to plasma protein of blood that makes neuronal cells occurring beyond blood brain barrier more vulnerable to oxidative and nitrosative stress in which DRG cells are the direct targets [2, 4]. The present study reveals that damage to these cells is in fact a precursor event during the execution of full-fledged CP-induced neurotoxicity. In such situation, CP firstly alters the cellular antioxidant and detoxifying systems that facilitates reactive oxygen free radicals to damage the biomolecules like DNA, proteins, and enzymes of the cell [13, 16]. As the dose increases, the extent of damage increases where nitrosative stress also joins oxidative stress that might even generate highly aggressive peroxynitrite radicals that might further engage cellular inflammatory response severing the histological integrity of the target region (Figure 1(b)). Besides, it has been documented that CP downregulates key antiapoptotic factors like Bcl-2 concomitant with upregulation of many apoptotic factors like caspase 3/7, activation of p53 that subsequently triggers MAPKs pathway to facilitate programmed cell death in the target cells including DRG cells [10, 20]. However, in the present work, it is quite possible that the repeated doses of CP elevate the oxidative and nitrosative stress up to the level that deteriorates the structural and functional integrity of DRG cells consequently leading to induction of necrosis instead apoptosis. This conception is well supported by Figure 2 where CP treated group demonstrated a significant rise in the number of necrotic cells as compared to the RF treated group. However, in the case of combination treated group IV, the DRG cells appear to be comparable to the control cell with relatively integrated histology. Earlier, it has been observed that both the compounds (RF and CP) interact with others* in vivo* to form four types of complex [16]. It is believed that this complex formation subsequently might not allow CP to invade on the cellular structure concomitant with ceasing oxidative and nitrosative stress to the threshold level that consequently favors apoptosis as mode of cell death over necrosis [16, 17]. Besides, the residual RF left after complex formation might be involved in the reorientation of energy releasing metabolism that might maintain cellular ATP level enough to facilitate apoptosis in contrary to the CP alone-treated group [16]. Moreover, RF has been documented to act as potential apoptosis inducer under the photoillumination condition like that in the present study [7, 10, 17, 21, 22]. Hence, downregulation of the stress with the sufficiency of energy with a higher propensity of RF as apoptosis inducer might orchestrate all the cellular, metabolic, and genetic factors at macro as well as the micro level that might trigger apoptosis in the combination treated group. Recently, Salman and Naseem [23] have successfully established the adjuvant property of RF to CP in cancer-induced mouse model based study in which the vitamin was able to enhance the anticancer activity of the drug along with a decrease in the drug-induced toxic insults to a significant extent. Its clinical trials on cancer patients are also ongoing, in which so far many encouraging outcomes have been observed.

Hence, the present investigation establishes the ameliorative effect of RF against CP-induced peripheral neuropathy that vividly demonstrates the propensity of RF to induce apoptosis in CP-based chemotherapy at the cost of lesser side effect. Therefore, RF should be recommended during CP-based chemoradiotherapy to realize the optimum clinical outcomes.

## Abbreviations

CP: Cis-Diamminedichloroplatinum II
RNS: Reactive nitrogen species
RF: Riboflavin
-SH: Sulfhydryl groups
BBB: Blood brain barrier.
